# Rapid Resolution of Anticipatory Vomiting in a Child With Chronic Illness: A Single-Session CBT Case

**DOI:** 10.1192/j.eurpsy.2026.11731

**Published:** 2026-07-31

**Authors:** N. Kapucu, Y. Çiçek, M. C. Tarakçıoğlu

**Affiliations:** 1 Child and Adolescent Psychiatry; 2 Psychiatry, Istanbul University- Cerrahpaşa, Istanbul, Türkiye

## Abstract

**Introduction:**

Behçet’s disease is a chronic multisystem inflammatory disorder that may present in childhood with recurrent oral ulcers, uveitis, and skin lesions (ITR-ICBD, 2014; Koné-Paut, 2016). A 9-year-old boy receiving intramuscular methotrexate for Behçet’s disease developed anticipatory nausea and vomiting triggered by injection cues. This case describes how a single session of cognitive behavioral therapy (CBT) rapidly reduced these conditioned symptoms and restored treatment adherence.

**Objectives:**

A 9-year-old boy with Behçet’s disease was referred to the child and adolescent psychiatry clinic due to recurrent nausea and vomiting associated with intramuscular methotrexate injections. He had been diagnosed three years earlier after recurrent uveitis episodes. Following several oral treatments, intramuscular methotrexate 20 mg weekly was initiated, along with colchicine 0.5 mg twice daily, achieving reasonable disease control.

Initially, he tolerated the injections without difficulty. About ten months before referral, mild nausea began to appear before injections and gradually worsened. In recent weeks, nausea started as soon as his mother took the medication box in her hand and escalated to severe vomiting during the injection itself. Symptoms subsided immediately afterward, suggesting a conditioned anticipatory response rather than a direct pharmacologic reaction.

**Methods:**

A single 45-minute CBT session was conducted. In this session, the injection was not performed; instead, the medication box was shown to elicit anticipatory cues safely. When nausea and discomfort intensified, the therapist applied breathing, relaxation, and short running exercises to demonstrate that these sensations were temporary and controllable. The patient enjoyed running, which was integrated as a behavioral activation technique to strengthen his sense of control. He remained cooperative and practiced the learned strategies effectively.

**Results:**

During the next injection, he delayed vomiting for several seconds; one week later, he was able to control the urge completely. Marked symptom reduction was achieved after a single CBT session, and follow-up sessions were recommended to reinforce coping and prevent relapse.

**Conclusions:**

This case illustrates that anticipatory nausea and vomiting related to intramuscular medication can occur in children with chronic illnesses. Cognitive behavioral therapy was associated with rapid symptom reduction after a single session and helped the patient regain control of the conditioned response. Early recognition and prompt behavioral intervention may prevent escalation of procedural anxiety and reduce distress during medical treatments.

**Disclosure of Interest:**

None Declared

